# A global optimisation approach to range-restricted survey calibration

**DOI:** 10.1007/s11222-017-9739-5

**Published:** 2017-03-21

**Authors:** Ferran Espuny-Pujol, Karyn Morrissey, Paul Williamson

**Affiliations:** 1 Health Economics Group, Norwich Medical School, University of East Anglia, Norwich, NR4 7TJ UK; 2 European Centre for Environment and Human Health, University of Exeter Medical School, Truro, UK; 3 Department of Geography and Planning, University of Liverpool, Liverpool, UK

**Keywords:** Calibration estimation, Calibration weighting, Design-based inference, Generalised regression, Penalised calibration, Raking, Ridge calibration, Range restrictions

## Abstract

Survey calibration methods modify minimally sample weights to satisfy domain-level benchmark constraints (BC), e.g. census totals. This allows exploitation of auxiliary information to improve the representativeness of sample data (addressing coverage limitations, non-response) and the quality of sample-based estimates of population parameters. Calibration methods may fail with samples presenting small/zero counts for some benchmark groups or when range restrictions (RR), such as positivity, are imposed to avoid unrealistic or extreme weights. User-defined modifications of BC/RR performed after encountering non-convergence allow little control on the solution, and penalisation approaches modelling infeasibility may not guarantee convergence. Paradoxically, this has led to underuse in calibration of highly disaggregated information, when available. We present an always-convergent flexible two-step global optimisation (GO) survey calibration approach. The feasibility of the calibration problem is assessed, and automatically controlled minimum errors in BC or changes in RR are allowed to guarantee convergence in advance, while preserving the good properties of calibration estimators. Modelling alternatives under different scenarios using various error/change and distance measures are formulated and discussed. The GO approach is validated by calibrating the weights of the 2012 Health Survey for England to a fine age–gender–region cross-tabulation (378 counts) from the 2011 Census in England and Wales.

**Electronic supplementary material:**

The online version of this article (doi:10.1007/s11222-017-9739-5) contains supplementary material, which is available to authorized users.

## Introduction

*Survey calibration* incorporates auxiliary information to a sample in two closely related ways: weighting and estimation. *Calibration weights* make a sample consistent with auxiliary information (e.g. census population totals) while in general respecting the initial sample design (Deville and Särndal 1992). Resulting *calibration estimates* of population parameters (e.g. totals) improve direct sample estimates (e.g. Horvitz–Thompson). Survey calibration methods can be also applied to adjust for non-response or coverage limitations, and to outlier detection (Särndal 2007). The internal consistency of administrative data can be intrinsically guaranteed with survey calibration, since it may provide a common degree of agreement between estimates from multiple samples of the same population (Wu and Lu 2016).

Calibration estimates were initially introduced for finite population totals or averages of either categorical or continuous variables. Example methods are the *generalised regression* (GREG) and *raking* estimators (Deville and Särndal 1992; Singh and Mohl 1996). Calibration estimates were later developed for variance and bilinear parameters (Théberge 1999), quantiles and ratios (Särndal 2007). Given an initial value for frequency tables with no zeroes, if either auxiliary cells or marginal counts are known, the corresponding *poststratification* problems can also be modelled using survey calibration (Deville and Särndal 1992). In particular, direct standardisation of rates (e.g. age–sex standardisation as commonly used in epidemiology and demography), can also be addressed using survey calibration (Lumley 2010).

Survey calibration methods search for (real-valued) calibration weights that: (i) satisfy a set of *benchmark constraints* (BC) or calibration equations and, in most cases, (ii) are close to initial weights. Therefore, calibration estimators are: (i) *design consistent* and (ii) (asymptotically) *design unbiased* (Deville and Särndal 1992; Fuller 2002; Särndal 2007). In general, the use of auxiliary information in form of BC allows for bias and/or variance reduction in population-level estimates. The bias in calibration estimators is kept small by staying close to initial (design) weights through the minimisation of a distance measure. Some calibration methods use distances with undesirable effects that are likely to inflate the bias and/or variance: GREG may produce negative weights, and raking extreme ones. Outliers, small domain estimation or the estimation of nonlinear population parameters are also likely to produce extreme and highly variable weights (Théberge 2000; Wu and Lu 2016). *Range restrictions* (RR) on weights are imposed in practice in order to avoid weights taking unrealistic or extreme values. These can be imposed directly on weights or through the function measuring the distance to initial weights (Singh and Mohl 1996).

Failure of survey calibration methods may occur with real data (Sautory 1991; Tanton et al. 2011). In fact, BC may have no exact solution (zero error), either considered solely or in combination with RR on weights (Singh and Mohl 1996). This can be due to: the sample not being representative (enough) of every non-void class in the cross-classified BC; the RR being too tight; the BC forming an inconsistent system of equations, due to their derivation from differing data sources or from data with added noise as a result of statistical disclosure control procedures (Tanton et al. 2011). In addition, calibration algorithms may be unstable for too many BC or when multi-collinear survey variables are being benchmarked (Sautory 1991; Rao and Singh 1997). The existence of a solution to the range-restricted survey calibration problem was studied theoretically in Théberge (2000).

Faced with non-convergence of standard algorithms in practice, alternative approaches have been proposed: non-modelling heuristics and penalised calibration, also known as ridge calibration. Heuristics typically used include: broadening the categorisation of benchmark variables, modifying the benchmarking values, loosening the RR on weights, or even deleting some BC (Sautory 1991; Bankier et al. 1992). In Tanton et al. (2011), an upper threshold on the *total absolute error* (TAE) in BC is used to accept non-convergent solutions satisfying RR, with no further control for error in BC. *Penalised calibration* allows a certain degree of relaxation in each BC, being controlled by costs (or tolerances), while still providing approximately unbiased and asymptotically design-consistent estimators. Penalised versions of GREG with RR can be found adaptively adjusting the set of tolerances on BC errors in Rao and Singh (1997) and addressing its global minimisation in Wagner (2013). Non-convergence is still reported by penalisation methods, being possible even for loose RR on weights.

This paper proposes a global optimisation (GO) two-step approach to range- restricted survey calibration. First, the problem feasibility is guaranteed by optimally modifying the BC and (for the first time) RR, if needed and in a controlled manner. Second, the (always-feasible) resulting calibration problem is solved. The GO method can provide asymptotically design-consistent and realistic solutions, avoiding non-convergence problems and thus overcoming the typical need for user-defined heuristics. Moreover, by keeping close to initial weights, GO is approximately unbiased when design weights are available, otherwise benefiting from the *a priori* information provided by initial weights.

In Sect. 2, we provide the technical formulations of range-restricted survey calibration and calibration-based estimation of totals and variance of estimates. We also summarise the existing (unsuccessful) “penalisation” attempts to address non-convergence. In Sect. 3, it is shown that the feasibility of a range-restricted calibration problem can be checked in advance by solving a (generally sparse) linear program. Moreover, using the $\ell _1$-norm as an error measure, it is shown that feasibility can be achieved if allowing for a minimum (TAE) error in BC and/or a minimum change in RR. In both cases, the formulations are sparse linear programming problems, which have the advantage of being efficiently solvable for many variables; $\ell _1$-norm penalisation for errors in addition allows modification of only a small number of benchmark totals or range restrictions. Alternative error functions and modelling options are also discussed.

In Sect. 4, the generic GO algorithm for an always-convergent globally optimal survey calibration is presented. The Chi-square distance as in GREG is used for demonstration, its minimisation being globally optimal, leading to approximately unbiased estimators. In fact, GREG-based methods allowing for RR on weights are a particular case of GO, provided that the earlier are convergent (to a global optimum). The applicability of GO in small domain estimation and microsimulation contexts is summarised in Sect. 5.

In Sect. 6, the performance of the GO method is exemplified with real data by calibrating the weights of the 2012 Health Survey for England to population totals from the 2011 Census in England and Wales. Broad age–gender (20 groups) and region (9 groups) total counts are imposed as exact BC, while optimally controlled errors are allowed to calibrate HSE weights to a fine age–gender–region cross-tabulation (378 counts). The resulting calibration weights are further used to estimate the total counts for a broad age by economic activity cross-tabulation, which is used for validation purposes.

*Notation* The symbols $\mathbf {1}$ and ${\mathbf {0}}$ will denote constant vectors with all components equal to 1 and 0, respectively. Comparison operators between two vectors will be used to denote componentwise comparisons, e.g. $\mathbf {1}\ge {\mathbf {0}}$.

## Background

Assume as given survey data corresponding to a sample *S* of size *n*, drawn from a population *U*, together with a *n*-dimensional vector $\mathbf {d}$ of survey weights (by default, but not limited to, the sample design weights). For $\mathbf {x}$, a *p*-dimensional vector of variables, assume as known the $n\times p$ survey matrix $\mathbf {X}$, which contains the values of $\mathbf {x}$ for all sample units. For simplicity, the totals for variables are considered as the measure of interest; the formulation is similar when using proportions or averages. The variables can be either continuous or categorical, the latter possibly expressed using indicator binary variables (1–0 valued) for each category group in order to exploit known total unit counts for that group. The Horvitz–Thompson direct survey estimate of the population totals for the values of $\mathbf {x}$ is $\mathbf {t}_\mathbf {x}^{HT} = \mathbf {X}'\mathbf {d}$ (Horvitz and Thompson 1952). Also assume as given a more precise estimate $\mathbf {t}_\mathbf {x}$ of the totals of $\mathbf {x}$ for the population, this estimate provided for example by administrative census sources.

### Survey calibration

Survey calibration aims at determining new survey weights $\mathbf {w}$ that make the survey compatible with the known auxiliary totals, i.e. satisfying the *benchmark constraints*:^1^

BC $$\begin{aligned} \mathbf {X}'\mathbf {w}-\mathbf {t}_\mathbf {x}= {\mathbf {0}}\;. \end{aligned}$$

The weights should realistically represent units: for example, counts of households or persons have to be positive (in general, $\mathbf \ell _1 \mathbf {1}\le \mathbf {w}\le u_1 \mathbf {1}$, for two constants $\mathbf \ell _1$, $u_1$). Moreover, a drastic change in any particular weight from its initial value should be avoided (in general, $\mathbf \ell _2 \mathbf {d}\le \mathbf {w}\le u_2 \mathbf {d}$, for two constants $\mathbf \ell _2$, $u_2$). Accordingly, the weights can be subject to *range restrictions* in the form

RR $$\begin{aligned} {\mathbf {l}}\le \mathbf {w}\le {\mathbf {u}}\;, \end{aligned}$$

being ${\mathbf {l}}$ and ${\mathbf {u}}$ known vectors.^2^ Finally, in order to lead to unbiased estimates, the weights should ideally respect as much as possible the set of initial weights $\mathbf {d}$, which is achieved by minimising a distance ${\mathscr {G}}_\mathbf {d}(\mathbf {w})$ between $\mathbf {w}$ and $\mathbf {d}$.

Therefore, the mathematical problem associated with *range-restricted survey calibration* reads:

1 $$\begin{aligned} \begin{array}{rll} \arg \min \nolimits _\mathbf {w}&{} {\mathscr {G}}_\mathbf {d}(\mathbf {w})&{}\\ s.t. &{} \mathbf {X}'\mathbf {w}-\mathbf {t}_\mathbf {x}= {\mathbf {0}} &{}\text {(BC)}\\ &{} {{\mathbf {l}}\le \mathbf {w}\le {\mathbf {u}}} &{}\text {(RR)} \end{array} \end{aligned}$$

being $\mathbf {w}$ the *calibration weights* searched for.^3^ The resulting weights are used to make the survey compatible with known auxiliary totals and in particular can be used to adjust to non-response or coverage errors.

Following (Deville and Särndal 1992), the function ${\mathscr {G}}_\mathbf {d}(\mathbf {w})$ is assumed to be, for every fixed $\mathbf {d}>{\mathbf {0}}$: nonnegative, differentiable, strictly convex, defined on an interval containing $\mathbf {d}$, such that ${\mathscr {G}}_\mathbf {d}(\mathbf {d})= 0$, and having a differential continuous and locally invertible at $\mathbf {d}$. A typical function ${\mathscr {G}}_\mathbf {d}$ for the distance to initial weights is the *modified Chi-square* or generalised least squares distance (Singh and Mohl 1996)^4^

2 $$\begin{aligned} {\mathscr {G}}_\mathbf {d}^\textit{GREG}(\mathbf {w})= \left( \mathbf {w}-\mathbf {d}\right) ' \mathbf {D}^{-1} \left( \mathbf {w}-\mathbf {d}\right) \;, \end{aligned}$$

where $\mathbf {D}={{\mathrm{diag}}}{(\mathbf {d})}$ is a diagonal matrix with the elements of $\mathbf {d}$ in the diagonal. In that case, the resolution of the survey calibration problem (1) if ignoring any range restrictions (RR) gives the *generalised regression* weights (Deville and Särndal 1992; Merkouris 2010):

3 $$\begin{aligned} \mathbf {w}^\textit{GREG} = \mathbf {d}+ \mathbf {D}\mathbf {X}\left( \mathbf {X}' \mathbf {D}\mathbf {X}\right) ^{-1} \left( \mathbf {t}_\mathbf {x}- \mathbf {X}'\mathbf {d}\right) \;, \end{aligned}$$

the ratio estimator weights being a particular case for $p=1$ if replacing $\mathbf {D}$ with ${{\mathrm{diag}}}(\mathbf {X})^{-1} \mathbf {D}$ (Deville and Särndal 1992).

Another commonly used distance function is the *modified discrimination information* associated with the raking estimator (Singh and Mohl 1996):

4 $$\begin{aligned} {\mathscr {G}}_\mathbf {d}^\textit{MDI}(\mathbf {w})= \sum _{i=1}^n \left( w_i \log {\left( \frac{w_i}{d_i}\right) } - w_i + d_i \right) \;. \end{aligned}$$

There is no explicit formula to obtain the raking weights, which, when ignoring (RR), have the form $\mathbf {w}^{MDI} = \mathbf {D}\exp (\mathbf {X}\varvec{\lambda })$, for $\varvec{\lambda }$ a *p*-dimensional vector (Lagrange multiplier) solution of $\mathbf {t}_\mathbf {x}= \mathbf {X}' \mathbf {D}\exp {\left( \mathbf {X}\varvec{\lambda }\right) }$ (Deville and Särndal 1992). The raking ratio algorithm in Deming and Stephan (1940) provided a solution for the particular case of contingency tables (poststratification), which translates into the benchmark variables being categorical group membership indicators, some linear combination/s of which is/are unity (i.e. the groups need not to be mutually exclusive) (Deville and Särndal 1992; Kott 2009).

### Calibration estimators of totals and variance

Assume additionally as given a $n\times r$ survey matrix $\mathbf {Y}$ containing the values of $\mathbf {y}$, a *r*-dimensional variable of interest, for all *n* sample units in *S*. The population totals $\mathbf {t}_\mathbf {y}$ for the variable $\mathbf {y}$ can be estimated using the direct Horwitz–Thompson estimator $\mathbf {Y}'\mathbf {d}$; however, the variance of this estimator is high. The calibration estimators make use of calibration weights $\mathbf {w}^{Cal}$, which account for available auxiliary information, to produce the new estimate

5 $$\begin{aligned} \mathbf {t}_\mathbf {y}^{Cal} = \mathbf {Y}' \mathbf {w}^{Cal}\;. \end{aligned}$$

In particular, calibration estimators are design-based, not making use of any regression model linking the target variable $\mathbf {y}$ with the auxiliary variables $\mathbf {x}$. Given that the considered calibration distances ${\mathscr {G}}_\mathbf {d}(\mathbf {w})$ satisfy the properties assumed in Sect. 2.1, calibration estimators are both asymptotically design unbiased and design consistent, all of them being asymptotically equivalent (Deville and Särndal 1992). Moreover, if the auxiliary information is sufficiently related to the variable $\mathbf {y}$, calibration estimators are more efficient than the Horvitz–Thompson estimator (Fuller 2002).

The variance of calibration estimators can be approximated asymptotically using the fact that all estimators are asymptotically equivalent to the generalised regression estimator $\mathbf {Y}' \mathbf {w}^{GREG}$ (Deville and Särndal 1992). A compact form for the asymptotic variance of the generalised regression estimator can be found e.g. in Merkouris (2010). Alternative jackknife estimates of variance can be used in a more general context and were shown to outperform Taylor-based techniques for estimating the variance of calibration estimators in Stukel et al. (1996). This paper will accordingly adopt a non-asymptotic jackknife approach to estimate the variance of calibration estimators, at the expense of a higher computational burden.

### Non-convergence and penalised calibration

Several iterative methods have been used to solve the survey calibration problem (1), when including (RR), for various distance functions (Singh and Mohl 1996). As explained in Introduction, there are many possible reasons for non-convergence, specially when considering RR in addition to BC. This is usually addressed by using heuristics that either modify the BC and/or RR or allow some error in (BC), but, however, do not perform any optimal control of that error.

*Penalised calibration* instead searches for weights satisfying (RR) while allowing parametrically for errors in (BC). This is done via the minimisation of a compromise between the distance to initial weights and the errors in BC:

6 $$\begin{aligned} {\mathscr {G}}_\mathbf {d}^{GREG}(\mathbf {w}) + \left( \mathbf {X}'\mathbf {w}-\mathbf {t}_\mathbf {x}\right) ' \varvec{\varLambda }^{-1} \left( \mathbf {X}'\mathbf {w}-\mathbf {t}_\mathbf {x}\right) \;, \end{aligned}$$

where $\varvec{\varLambda } = {{\mathrm{diag}}}( \varvec{\lambda } )$ is a diagonal matrix depending on parameters $\varvec{\lambda } = (\lambda _j), 1\le j \le p$. The smallest possible values for these parameters are iteratively searched for in Rao and Singh (1997), where in fact these are obtained as a function of user-specified tolerances on the errors in (BC). Although this approach is shown to reduce the discrepancy in respecting (BC) for given (RR), its dependence on parameters used to control for errors in (BC) is critical for convergence. See e.g. Théberge (2000) for a closed-form solution to the problem, and Beaumont and Bocci (2008) and Section 9 in Fuller (2002) for closely related model-based “ridge regression” approaches. See Chen et al. (2002) for a less general modelling of errors using empirical likelihood methods.

Similarly, in Wagner (2013) a vector of unknowns $\varvec{\varepsilon }_B$ was used to model the multiplicative error in part of the benchmark totals so that the corresponding subset $B$ of BC is satisfied: $\mathbf {X}_B'\mathbf {w}= {{\mathrm{diag}}}( \mathbf {t}_{\mathbf {x},\,B}) \varvec{\varepsilon }_B$. Even if this approach required no “penalisation” parameters, convergence problems arose in a simulation even if allowing any value for the errors $\varvec{\varepsilon }_B$ in some BC, and increased significantly (14–23% failure) when imposing actual bounds on those errors.

## Optimal control for RR and BC to allow successful range-restricted calibration

All existing methods addressing the range-restricted survey calibration problem (1) run into non-convergence issues or lack of control of the errors in BC, even if using penalisation formulations like (6) that theoretically allow for the minimum error in BC. The usual approach consists in running a calibration method and at the end (after a long running time), if encountering non-convergence, requires an user to adjust the RR and/or BC. We propose instead to assess if the given values for RR and BC allow the existence for a solution (*feasibility*), and to compute optimal alternative values that guarantee the feasibility in case of foreseen non-convergence, given user-specified values for the tolerance on errors in BC, and binding and loose values for RR.

The natural questions that we will address in this section are, given RR vectors ${\mathbf {l}}, {\mathbf {u}}$:^5^ is problem (1) feasible? is it feasible if allowing a certain error $\varepsilon$ in BC? in fact, what is the minimum error that needs to be allowed in BC to achieve feasibility? alternatively, what is the smallest change in RR that we need to perform to obtain feasibility (even if possibly allowing for some error $\varepsilon$ in BC)?

### An introductory example

Before entering into details, let us inspect the previous questions in a very simple scenario with $n=100$ individuals in a sample with initial weights $\mathbf {d}=20 \cdot \mathbf {1}$ to be calibrated using one known benchmark constraint BC:

7 $$\begin{aligned} w_1 + \dots + w_{100} = 2016 \end{aligned}$$

If we impose as RR the positivity of weights ${\mathbf {0}} \le \mathbf {w}$, the BC and RR can be satisfied simultaneously, e.g. by setting all weights equal to 20.16, or by setting 99 weights to 20 and just one weight to 36 (the calibration solution will depend on the distance function used to measure changes in initial weights). However, if we impose that ${\mathbf {0}} \le \mathbf {w}\le 20 \cdot \mathbf {1}$ as RR on weights in order to avoid any survey unit to represent more than 20 total units, the BC (7) cannot be satisfied; thus, the combination of BC and RR is incompatible. In that case, if we allow a small arbitrary error of 100 in (7), the problem becomes feasible by taking all weights equal to 20. In doing so, we obtain an error in BC equal to 16, which is in fact the minimum needed for compatibility with the given RR. Alternatively, the upper bound on weights can be set to ${20.16}\cdot \mathbf {1}$ (or any higher value) in order to have feasibility given the original BC.

### Can the problem be solved?

The existence of a solution (*feasibility*) for the range-restricted calibration problem (1) is equivalent to the existence of solutions for the set of constraints:

8 $$\begin{aligned} \left\{ \begin{array}{l} \mathbf {X}'\mathbf {w}-\mathbf {t}_\mathbf {x}= {\mathbf {0}}\,,\\ {{\mathbf {l}}\le \mathbf {w}\le {\mathbf {u}}}\;. \end{array} \right. \end{aligned}$$

If this set is non-void, then it will be possible to find in it the vector of weights $\mathbf {w}$ at minimum distance ${\mathscr {G}}_\mathbf {d}(\mathbf {w})$ to the set of initial weights $\mathbf {d}$. By expressing the equality as two inequalities, the system (8) can be seen as a set of affine inequalities in the unknown $\mathbf {w}$, and therefore, its feasibility could be checked using direct methods like system reduction by repeatedly using Fourier–Motzkin elimination (Dantzig and Eaves 1973). An alternative algorithm for assessing the existence of a solution to the range-restricted calibration problem was developed in Théberge (2000). However, the existence of a solution can also be addressed by computing the minimum change needed in BC or RR for feasibility (following sections): if no change is needed, then the original problem is feasible; otherwise, the minimum needed change has already been computed.

### Feasibility guarantee allowing minimum error in BC

The minimum *total absolute error* (TAE) in BC needed for their compatibility with the given RR is

9 $$\begin{aligned} \begin{array}{rl} TAE^*= \min \nolimits _\mathbf {w}&{} \Vert \mathbf {X}' \mathbf {w}- \mathbf {t}_\mathbf {x}\Vert _1 \\ s.t. &{} {{\mathbf {l}}\le \mathbf {w}\le {\mathbf {u}}}\;, \end{array} \end{aligned}$$

being $\left\| \mathbf {v}\right\| _1$ the $\ell _1$-norm of a *p*-dimensional vector $\mathbf {v}$, defined by $\left\| \mathbf {v}\right\| _1 = \sum _{i=1}^p | v_i |$. The total absolute error has a very easy physical interpretation given that its units are the same as those of the population totals $\mathbf {t}_\mathbf {x}$. The minimisation of the TAE error can be written as the minimisation of $\Vert \varvec{{\tilde{\varepsilon }}}\Vert _1= \varvec{1}'\;\varvec{{\tilde{\varepsilon }}} = {\tilde{\varepsilon }}_1 + \dots + {\tilde{\varepsilon }}_p$, for a nonnegative vector $\varvec{{\tilde{\varepsilon }}}$ such that $| \mathbf {X}'\mathbf {w}-\mathbf {t}_\mathbf {x}| \le \varvec{{\tilde{\varepsilon }}}$ (componentwise). By decomposing the absolute value, we obtain two vector inequalities,^6^ and therefore:

10 $$\begin{aligned} \begin{array}{rl} TAE^*= \min \nolimits _{\mathbf {w},\,\varvec{{\tilde{\varepsilon }}}} &{} \varvec{1}'\;\varvec{\tilde{\varepsilon }} \\ s.t.&{} \mathbf {X}'\mathbf {w}- \varvec{\tilde{\varepsilon }}\le \mathbf {t}_\mathbf {x}\;,\ -\mathbf {X}'\mathbf {w}-\varvec{\tilde{\varepsilon }} \le - \mathbf {t}_\mathbf {x}\;,\\ &{} {{\mathbf {l}}\le \mathbf {w}\le {\mathbf {u}}}\;,\ \varvec{\tilde{\varepsilon }} \ge {\mathbf {0}} \;. \end{array} \end{aligned}$$

Given that both the objective and all constraint functions are affine in the unknowns $({\mathbf {w},\varvec{\tilde{\varepsilon }}})$, we have shown that finding the optimal TAE is a linear programming problem (Boyd and Vandenberghe 2004). This class of convex optimisation problems may be solved quickly and with global optimality convergence guaranteed by exploiting duality relations and optimality theorems (Boyd and Vandenberghe 2004). If the solution is $TAE^*=0$, it means that the calibration problem (1) is feasible; nonzero minimum TAE values require the modification of the original problem as proposed in Sect. 4.

### Feasibility search by allowing minimum change in RR and user-specified error in BC

Assume now that we do not want to have a TAE error in BC greater than a scalar value $\varepsilon$ (ideally equal to 0), but that we allow a small change in RR provided by two nonnegative vectors $\varvec{\lambda },\varvec{\mu }$, while keeping the weights inside a limiting range: ${\mathbf {L}}\le \mathbf {w}\le {\mathbf {U}}$. The smallest possible *total absolute change* (TAC) in RR that guarantees feasibility with TAE error below $\varepsilon$ and final weights inside the maximum limiting range, if it exists, can be computed as

11 $$\begin{aligned} \begin{array}{rl} TAC^* = \min \nolimits _{\varvec{\lambda },\,\varvec{\mu },\, \mathbf {w}} &{} \Vert \varvec{\lambda }\Vert _1 + \Vert \varvec{\mu }\Vert _1 \\ s.t. &{} \Vert \mathbf {X}' \mathbf {w}- \mathbf {t}_\mathbf {x}\Vert _1 \le \varepsilon \;, \\ &{} {{\mathbf {L}}\le {\mathbf {l}}- \varvec{\lambda } \le \mathbf {w}\le {\mathbf {u}}+ \varvec{\mu } \le {\mathbf {U}}}\;,\ \\ &{}\varvec{\lambda }\ge \varvec{0}\;,\ \varvec{\mu } \ge \varvec{0}\;. \end{array} \end{aligned}$$

The associated linear programming problem reads:

12 $$\begin{aligned} \begin{array}{rl} TAC^* = \min \nolimits _{\varvec{\lambda },\,\varvec{\mu },\, \mathbf {w},\, \varvec{\tilde{\varepsilon }}} &{} \varvec{1}'\;\varvec{\lambda } + \varvec{1}'\;\varvec{\mu } \\ s.t. &{} \mathbf {X}'\mathbf {w}- \varvec{\tilde{\varepsilon }}\le \mathbf {t}_\mathbf {x}\;,\ -\mathbf {X}'\mathbf {w}-\varvec{\tilde{\varepsilon }} \le - \mathbf {t}_\mathbf {x}\;,\ \varvec{1}'\;\varvec{\tilde{\varepsilon }} \le \varepsilon ,\\ &{} -\mathbf {w}- \varvec{\lambda } \le -{\mathbf {l}}\;,\ \mathbf {w}- \varvec{\mu } \le {\mathbf {u}}\;,\\ &{}\ \varvec{0} \le \varvec{\lambda } \le {\mathbf {l}}- {\mathbf {L}}\;,\ \varvec{0} \le \varvec{\mu } \le {\mathbf {U}}- {\mathbf {u}}\;,\ \varvec{\tilde{\varepsilon }} \ge {\mathbf {0}}\;. \end{array}\nonumber \\ \end{aligned}$$

This problem has the trivial solution $$TAC^*=0$$ for values $$\varepsilon $$ above or equal to $$TAE^*$$, given that TAE* is the minimum error needed without modifying the RR. It may happen that the problem has no solution for a given $$\varepsilon $$ smaller than $$TAE^*$$, for instance for $$\varepsilon =0$$ with non-consistent BC. More precisely, the problem (12) will be feasible only for values of $$\varepsilon $$ above or equal to $$TAE^{**}$$, where TAE** is the minimum error needed modifying the RR with the limit bounds $${\mathbf {L}}\le \mathbf {w}\le {\mathbf {U}}$$. Errors smaller than TAE** cannot be obtained; the solution of (12) with $$\varepsilon =$$TAE** will have TAC*$$\le \varvec{1}'\left( {\mathbf {l}}- {\mathbf {L}}\right) + \varvec{1}'\left( {\mathbf {U}}- {\mathbf {u}}\right) $$, the later being the biggest possible change in RR. Values of $$\varepsilon $$ between TAE** and TAE* will have a solution $$TAC^*>0$$, so the original calibration problem (1) will need to be modified to be feasible, as proposed in Sect. 4.

### Alternative possibilities to model feasibility

The global optimality and simplicity of the previous approach are not affected if constant factors are introduced. For example, different relative weights $\varvec{r}$ may be assigned to different BC, by just replacing in (10) and (12) the expression $\varvec{1}'\varvec{\tilde{\varepsilon }}$ with $\varvec{r}'\varvec{\tilde{\varepsilon }}$. In particular, in the case that the benchmark values are provided from administrative totals of cross-tabulated variables, it is possible to normalise the TAE by dividing each benchmark constraint by the relevant total of the corresponding administrative table, so that the global measure of error is a sum of comparable errors. Similarly, it is possible to divide the benchmark totals $\mathbf {t}_\mathbf {x}$ by the relevant benchmark table totals $\varvec{v}$ so that the calibration weights represent proportions, by just replacing $\mathbf {t}_\mathbf {x}$ with ${{\mathrm{diag}}}(\varvec{v})^{-1}\mathbf {t}_\mathbf {x}$.

Different precision levels on BC can be also achieved, e.g. having some “exact” BC as in Wagner (2013) or some BC with a smaller penalisation to errors as in Rao and Singh (1997), by setting in (10) and (12) the corresponding components of $\varvec{\tilde{\varepsilon }}$ to the desired precision values. This option will be used in the experimental validation of the paper, where the exact BC will correspond to a broad cross-tabulation and a finer cross-tabulation will be used as inexact BC. If wanting to add a “Gelman” bound $\kappa _ GB$ to control for the ratio of the largest to the smallest calibrated weight, two scalar variables $\alpha \ge 0\,,\ \beta \ge 0$ and the linear constraints $\{ \alpha \mathbf {1}\le \mathbf {w}\le \beta \mathbf {1}\,,\; - \kappa _ GB \alpha + \beta \le 0\,\}$ need to be added to the optimisation programs (Wagner 2013). The modified problems remain linear given that these constraints are linear. The problem in Sect. 3.4 can usually be simplified: if the lower bound ${\mathbf {l}}$ has to be ${\mathbf {0}}$ (positivity of weights), then only the upper bound can be varied, which can be done by only estimating $\varvec{\mu }$ while setting $\varvec{\lambda }$ to zero; or if the changes can be the same for all RR, then only one parameter needs to be used for each of the increment vectors $\varvec{\lambda }$ and $\varvec{\mu }$.

The ideal choice of a penalisation function should be based on distributional assumptions for errors in BC and desired changes in RR,^7^ depending on the problem and available computational power. The proposed $\ell _1$-norm allows a linear programming implementation and is a robust penalisation that in practice produces many very small residuals, allowing to identify many BC that can be satisfied exactly e.g. by looking at the components of $\varvec{\tilde{\varepsilon }}$ in (10). A simpler approach can use the $\ell _\infty$-norm to penalise errors (the maximum component in a vector being penalised), which is equivalent to considering as single-valued the unknown vectors $\varvec{\tilde{\varepsilon }}$ in (10) and $\varvec{\lambda },\varvec{\mu }$ in (12).

Using a $\ell _2$-norm or a weighted least squares penalisation converts the feasibility programs into quadratic and quadratically constrained quadratic, which are solvable for less variables and are more time-consuming than linear programs (Boyd and Vandenberghe 2004). In fact, Théberge proposed using a quadratic norm to allow minimum errors in BC for GREG without RR in Théberge (1999) (a closed-form solution exists). In Théberge (2000), he further formulated the problem with RR (Section 2) but adopted a not always-convergent penalisation-like formulation for its resolution (Section 4).

## Optimally modelling and solving range-restricted survey calibration

We have seen in Sect. 3 that the feasibility of the range-restricted calibration problem (1) has linear complexity, independently of the chosen distance function $\mathscr {G}_\mathbf {d}(\mathbf {w})$, and it can be achieved with equal complexity level by allowing optimally controlled errors in BC and/or changes in RR. In this section, we propose Algorithm 1 to solve the range-restricted survey calibration problem (1) for any given RR bounds by allowing the minimum needed modification(s) of the BC tolerance. A more complex Algorithm 2 is also proposed, automatically performing optimal modification(s) of RR, only if needed and controlling for the error in BC via a user-defined parameter $\varepsilon$. We also discuss the choice of a distance function ${\mathscr {G}}_\mathbf {d}(\mathbf {w})$ in the proposed algorithms and focus on the particular Chi-square distance for demonstration.
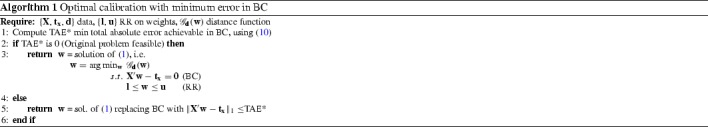


Algorithm 1 solves the range-restricted calibration problem (1) in two steps: first, the $TAE^*$ minimum value is computed; then, the calibration problem allowing TAE error in BC equal to $TAE^*$ is solved optimally. The convergence of this two-step approach is guaranteed by construction while respecting the asymptotic design consistency. The advantage with respect to existing penalisation methods is that feasibility is optimally guaranteed in advance by solving a simple linear program, without need to test the convergence of any calibration problem.

In practice, while it is usually plausible to agree in some limiting bounds ${\mathbf {L}}, {\mathbf {U}}$ for the value of weights, the choice of the RR values ${\mathbf {l}}, {\mathbf {u}}$ is somewhat arbitrary. For instance, if the units are persons or households weights must be positive (${\mathbf {L}}= {\mathbf {0}}$), but it could also be desirable to impose a minimum weight ${\mathbf {l}}>{\mathbf {0}}$ for each unit, its actual value being flexible, ideally allowing both for convergence and for a low error in BC if using Algorithm 1. This is the case both for RR expressed in absolute terms or as relative changes in initial weights, as justified in Sect. 2.1. Rather than manually exploring a range of possible RR values (best possible existing practice), Algorithm 2 optimally modifies RR given user-specified values $\varepsilon$ for the maximum allowed error in BC and ${\mathbf {L}}, {\mathbf {U}}$ as bounds for the final RR.

For sufficiently high values of $$\varepsilon $$, the tolerance to error in BC (infinity in the extreme case), Algorithm 2 will preserve the initial RR and return the same solution as Algorithm 1. In other words, Algorithm 1 can be obtained from Algorithm 2 by setting $$\varepsilon $$ to infinity. For values of $$\varepsilon $$ smaller than $$TAE^*$$, a further step is performed trying to achieve error in BC below $$\varepsilon $$ by modifying the RR with a minimum total absolute change $$TAC^*$$, always keeping the weights inside the limiting bounds $${\mathbf {L}}, {\mathbf {U}}$$. In case the modification of RR cannot lead to a feasible problem, Algorithm 2 proposes to use $$\varepsilon =TAE^{**}$$, the minimum possible error in BC for the given hard bounds $${\mathbf {L}}, {\mathbf {U}}$$ on RR.
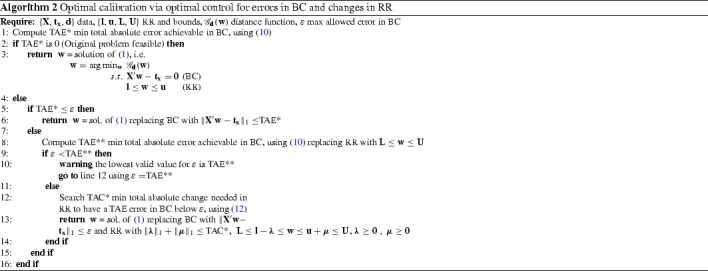


In both Algorithm 1 and Algorithm 2, the original range-restricted calibration problem (1) is solved if it is feasible (line 3), and otherwise, it is replaced by a problem of the form

13 $$\begin{aligned} \begin{array}{rl} \arg \min \nolimits _{\mathbf {w},\varvec{y}} &{} {\mathscr {G}}_\mathbf {d}(\mathbf {w})\\ s.t. &{} \varvec{A} \left( \begin{array}{l} \mathbf {w}\\ \varvec{y} \end{array}\right) \le \varvec{a}\;, \\ &{} {{\mathbf {b}} \le \left( \begin{array}{l} \mathbf {w}\\ \varvec{y} \end{array}\right) \le {\mathbf {c}}}\;, \end{array} \end{aligned}$$

for a matrix $\varvec{A}$, vectors $\varvec{a}$, ${\mathbf {b}}$, ${\mathbf {c}}$, and auxiliary variables $\varvec{y}$ defined by the algorithm. More specifically, the optimisation domain from (1) is modified in Algorithm 2 on line 6 (line 5 in Algorithm 1) by replacing the BC with $\Vert \mathbf {X}'\mathbf {w}-\mathbf {t}_\mathbf {x}\Vert _1 \le$ TAE*, and on line 10 in Algorithm 2 additional constraints of similar nature are added. The resulting domains can be expressed using affine inequalities in the form (13) with the help of auxiliary variables, as done in Sect. 3. Therefore, the complexity of the resulting problems will be mainly associated with that of the distance function ${\mathscr {G}}_\mathbf {d}(\mathbf {w})$.

Under the assumptions of Sect. 2.1 for the distance function ${\mathscr {G}}_\mathbf {d}(\mathbf {w})$, the problems (13) are convex with smooth objective and therefore can be solved with global optimality convergence guarantees by exploiting duality relations and optimality theorems like the necessity and sufficiency of Karush–Kuhn–Tucker conditions (Boyd and Vandenberghe 2004). The resolution can be done with fast convergence in particular cases; a closely related example is the semismooth Newton method proposed in Wagner (2013) for the Chi-square distance (2) and the raking distance (4). Note that despite its possible efficient minimisation, a $\ell _1$ distance function is not suitable since it would allow a few weights being very distant from initial ones, which could undesirably cause a high bias in calibration estimators. Rather than developing resolution methods for different distances, this paper has focussed on developing a flexible always-convergent optimal calibration framework, which is exemplified by adapting the range-restricted generalised regression (GREG) estimator.

The range-restricted calibration problem (1) for the Chi-square distance (2) was identified as a quadratic programming problem in (Isaki et al. 2000), and its fast optimal resolution exploiting duality principles was addressed recently (Wagner 2013); however, its feasibility has not yet been guaranteed by any method. If using Algorithm 2 for this purpose, the resulting modified problems (13) are inequality-constrained quadratic convex optimisation programming problems. These can be solved in polynomial time, and in practice relatively quickly, while assessing the global optimality of the solution (Boyd and Vandenberghe 2004). Implementation details and source code for the simpler version in Algorithm 1 are provided in Appendix 1.

## Small domain estimation and microsimulation

When wanting to produce estimators for a small domain of the population, it is no longer efficient to use calibration weights that were computed using the whole survey and auxiliary totals for the whole population. Survey calibration at the domain level and/or knowledge on the domain size, or combining information from multiple surveys at the domain level, provides approximately unbiased design-consistent estimators with substantial variance reduction with respect to other estimators (Merkouris 2010). An intermediate option is adopted in small area estimation (by, for example, but not limited to, spatial microsimulation) when (sufficient) survey data are not available for a small area, consisting in using out-of-area survey data in combination with known area totals (Tanton 2014).

More in general, microsimulation may combine surveys and benchmarks corresponding to different periods of time or to non-exactly matching domains or areas (Ballas and Clarke 2009; Tanton 2014) or may calibrate survey-based but otherwise simulated data (Pudney and Sutherland 1994; Wittenberg et al. 2011). From a modelling perspective, reweighing survey data to match small area counts requires allowing for errors in BC. From a computing perspective, although computing power has exponentially improved, the problems of small area level estimation/production of small area level data are still computationally intensive. Our global optimisation approach automatically models and controls for errors in BC, and offers a computationally efficient method of producing small area level data by solving linear and quadratic optimisation programs.

## Evaluation

The proposed methods are demonstrated and validated using real datasets: the Health Survey for England 2012 (HSE) and the 2011 Census in England and Wales (CEW). The HSE is representative of the English population living in private households (Craig and Mindell 2012), and it is drawn in 2011 using a multi-stage stratified sampling approach. Available survey weights adjust for selection, non-response, and population age–gender and strategic health authority region profiles. CEW tables DC1104EW and DC1602EWLA provide population total counts for non-institutional residents in England. The initial survey sample for the experiments consisted of 10,308 individuals from HSE 2012.^8^

In all experiments,^9^ census-based population totals for 10 age groups cross-tabulated with gender and population totals for the 9 regions in England (20 + 9 counts for a population of size *N* = 52,059,931) were imposed as exact constraints, and the positivity of calibration weights was imposed as part of range restrictions (RR) on weights. An additional BC was imposed with 378 population totals for 21 age groups cross-tabulated with gender and region.^10^ This fine cross-tabulation was not available at the time of release of the HSE data. The experiments did not use any continuous benchmarks for the sake of simplicity, but benchmarks on continuous data, e.g. average age per region, could be incorporated.^11^ Population totals for five age groups by four economic activity groups (in-employment, ILO unemployed, retired, and other inactive) were used only for validation, but not as BC. All used BC and validation tables are provided as supplementary material.

Independently of any RR choice (or the initial sample weights), the fine age–gender–region cross-tabulation with 378 group totals cannot be satisfied exactly by calibration weights, given that the sample has a zero count for one group.^12^ Further motivated by the presence of some small counts, traditional calibration would only impose a broad cross-tabulation on the survey weights like the 29 age–gender and region counts that we will impose exactly in all experiments. However, the fine age–gender–region counts provide a much richer picture of the joint distribution of those variables, and the original weights are distant from correctly representing that picture: the total absolute error (TAE) of the HSE 2012 weights for that BC is of 7,858,083 units (a 15.14% of the total population size). Given that the cross-tabulation is categorical, the total absolute error is counting the number of individuals wrongly assigned to each cross-tabulation group. Since we are imposing exact broad age–gender population counts, the estimated population total remains fixed. Thus half of the TAE is the number of individuals being misclassified, which for the HSE estimate is 7.5% of the English population.

Instead of ignoring the fine age–gender–region cross-tabulation, it can be used to calibrate the sample weights if we allow some error in BC (which arises as a natural need for the given data), consequently resulting in better estimates on age–gender–region-related variables. In order to allow a direct comparison of the gain in adding this strategy to the traditional approach, we imposed as exact the already described broad age–gender and region cross-tabulations. We performed three validation experiments returning positive weights ($\mathbf {w}\ge {\mathbf {0}}$) at minimum Chi-square distance (2) to the initial sample weights:

**Ex1.**: Minimum TAE error in BC;
**Ex2.**: Minimum TAE in BC and $0.5 \cdot \mathbf {1}\le \mathbf {w}\le 3.5 \cdot \mathbf {1}$ as RR;
**Ex3.**: Minimum TAC change in Ex2 RR so that TAE $\le 0.1$%.

The corresponding resolution using optimisation programs is summarised in Algorithm 1 for Ex1 and Ex2, and in Algorithm 2 for Ex3. Implementation details and source code for Algorithm 1 are provided in Appendix 1.

For all experiments and for the original HSE data, we show in Table 1 the modified Chi-squared distance (2) between the HSE and each considered set of weights and provide descriptive statistics of the latter. Jackknife standard deviation (SD) estimates for the BC/validation count estimates were obtained using 94 groups of primary sampling units (PSUs), built up by deleting six PSUs at each time as described in Kott 1998. The error measures used for estimated counts were: TAE the total absolute error, the TAE as a percentage of the population total, TRE the total relative error (sum of relative errors over all group counts, as a %), and RMSE the root mean square error. Table 2 shows the average SD and fitting errors for the fine age–gender–region BC cross-tabulation estimates and Table 3 the average SD and errors for the age by economic activity validation count estimates.

**Table 1:** Chi-square distance and distribution statistics for the HSE and obtained calibration weights

	Chi-sq	Min	Q1	Median	Q3	Max	Max/Min
HSE	0.0	0.36	0.77	0.91	1.14	6.67	18.32
Ex1	570.3	0.31	0.73	0.90	1.14	7.26	23.31
Ex2	524.6	0.50	0.73	0.90	1.14	3.50	7.00
Ex3	549.1	0.36	0.73	0.90	1.14	5.04	14.00

**Table 2:** Age–gender–region cross-tabulation estimation (378 counts): average standard deviation (SD) over all estimates, and BC fitting errors

	SD	TAE	TAE (%)	TRE (%)	RMSE
HSE	205,835.4	7,858,083.0	15.09	6829.8	27,415.5
Ex1	3091.3	29,678.0	0.06	121.0	1079.4
Ex2	11,298.3	339,509.2	0.65	604.1	3227.8
Ex3	3525.2	52,059.9	0.10	192.3	1177.1

**Table 3:** Age by economic activity estimation (20 counts): average standard deviation (SD) over all estimates, and validation errors

	SD	TAE	TAE (%)	TRE (%)	RMSE
HSE	1,027,677.0	2,059,120.9	4.89	633.1	165,721.7
Ex1	861,528.8	1,763,839.5	4.19	494.1	130,193.5
Ex2	869,717.6	1,786,060.9	4.24	497.3	130,407.6
Ex3	856,300.1	1,749,775.5	4.16	492.8	129,310.1

Experiments Ex1–Ex3 only control for errors in BC, but do not use the validation table for calibrating the survey weights

The original HSE weights (HSE, first row in all tables) do not present very extreme values, the highest ratio between weights being 18.32 (Table 1). As already explained, the HSE weights perform badly in estimating the fine age–gender–region distribution in England: 15% of total absolute error and 6,829.8% of total relative error, with an average SD equal to 0.95% of the total population (Table 2). The broad age by economic activity cross-tabulation is quite well estimated by HSE with TAE error below 5% (Table 3). This is possibly in part because the broad categorisation of age is similar to the age categorisation which the HSE weights adjust for. See Appendix 2 for further discussion.

The Ex1 calibration weights (Ex1, second row in all tables) are at average Chi-square distance of 0.5 to the HSE weights and have slightly more extreme values, the highest ratio between weights being 23.31 (Table 1). The resulting Ex1 age–gender–region BC count estimates have much smaller SD than the HSE estimates, the proposed method being therefore more efficient, and have by construction a very small bias: the minimum possible TAE error in BC (Table 2). All considered SD and error indicators indicate consistently an improvement in performance when applying the Ex1 calibration weights to estimate the non-fitted validation totals (Table 3).

Experiment Ex2 (third row in all tables) provides an example of a practice commonly followed by practitioners and found in the literature, see e.g. Singh and Mohl (1996), Stukel et al. (1996), consisting in the arbitrary selection of range restriction values for the calibration weights and *a posteriori* observation of errors (in case of convergence). The Ex2 selected RR values result in a (user-defined) low dispersion in Ex2 weights (highest ratio between weights being 7), which computation required a minimum TAE error in BC of 0.65% for convergence. As a result the average deviances and all the fitting errors and validation errors are higher for Ex2 than those for the Ex1 weights (Tables 2 and 3). So far we have seen that Ex1 provided an optimal fit of the BC but at the expense of slightly more extreme weights, and also that an arbitrary choice of RR on weights in Ex2 achieved more centred weights at expense of increasing the SD and the (minimum) errors in both the BC and validation estimates.

It would certainly be time-consuming to perform an exploration of possible values for RR in order to obtain satisfactory weights with non-extreme values and low SD and low (minimum) fitting errors for the BC. Instead, Ex3 (fourth row in all tables) searches for the minimum change in provided initial values for RR at expense of allowing a (user-specified) 0.1% TAE error in fitting the fine age–gender–region BC counts. Compared with Ex2, both fitting and validation errors were smaller for Ex3. Compared with Ex1, Ex3 resulted in a set of weights with less extreme values, the highest ratio between weights being 14.00, and an small increase in (controlled) bias and SD in fitting the BC. Nonetheless, Ex3 provided (slightly) better estimates of the validation counts, pointing at a possible dangerous over-fitting effect if using the Ex1 approach: fitting too closely a fine cross-tabulation (having small counts) may well increase the bias and variance in estimation for non-fitted variables. However, in the case considered here, this effect was tiny compared to the efficiency gain and bias reduction with respect to estimates obtained using the initial HSE weights.

Overall, the three experiments Ex1–Ex3 used Algorithm 2 to minimally modify the HSE weights to adjust them to the fine age–gender–region BC cross-tabulation totals, overcoming the fact that the survey sample had small and even zero counts for that cross-tabulation. This was done by allowing for a minimum error in BC, which can be seen as equivalent to clustering some benchmark groups. Thus the experiments optimally improved a practice often followed arbitrarily to avoid non-convergence. In all experiments, not only was the fitted BC age–gender–region distribution much better approximated than with the original HSE weights, but also efficiency and performance improved when estimating age by economic activity validation total counts.

## Summary and conclusion

This paper has presented a two-step global optimisation (GO) approach to design-based survey calibration with guaranteed convergence, allowing for range restrictions on weights while controlling for those range restrictions and the (minimum) error in benchmark constraints.

First, GO assesses the feasibility of the range-restricted calibration problem, with infeasible problems being transformed into feasible ones by allowing minimal errors in the benchmark constraints (BC) and/or minimal changes in the weights’ range restrictions (RR). For this purpose, GO identifies the minimum achievable difference between the calibrated (reweighted) survey and the benchmark totals, taking into account any RR specified for the solution weights. It also identifies the minimum needed change in those RR, allowing exploration of an alternative solution, more respectful of the original problem, having zero or below-minimum error in BC (in general, the existence of solution is only guaranteed if allowing the minimum error in BC). All the problems involved in this first step assessing/guaranteeing feasibility are modelled using the robust $\ell _1$-norm penalisation ($\ell _\infty$- and $\ell _2$-norm alternatives, as well as weighted versions, were discussed in the text) and as a result can be solved efficiently using sparse linear programming. Second, the GO approach applies global optimisation techniques for minimising the change in weights subject to allowing only the minimum error in BC or change in RR required for feasibility (already computed in the previous step). The approach has been theoretically exemplified with the Chi-square distance being used to measure the change in weights with respect to initial (design) ones. Other distances have been considered, for which modern optimisation techniques will be useful to solve the resulting calibration problems.

The first step to assess/achieve feasibility represents an efficient modelling alternative to the current approaches in which convergence is known only after running a calibration method (time costly) and the reasons for non-convergence are not always clear. Moreover, existing approaches either make use of heuristics after encountering non-convergence, which do not offer enough control on the solution, or require user-defined parameters to model infeasibility, which in practice may not avoid non-convergence.

For survey calibration problems where the BC can be met, GO will provide a solution equivalent to that produced by calibration methods that allow RR on weights (assuming the chosen number of iterations in iterative methods poses no limit to convergence). GO-based estimators preserve the good properties of survey calibration estimators, design consistency, and asymptotic design unbiasedness, while adding guaranteed convergence and global optimality. In a real data experiment, we showed a double-win situation (gain in both bias and variance), achieved through two-level calibration: broad group cross-tabulations were imposed exactly, whereas a small group cross-tabulation (leading to zero counts in the survey) was managed optimally using the proposed approach.

### Electronic supplementary material

Below is the link to the electronic supplementary material.

Supplementary material 1 (pdf 43 KB)

## Figures and Tables

**Fig. 1:**
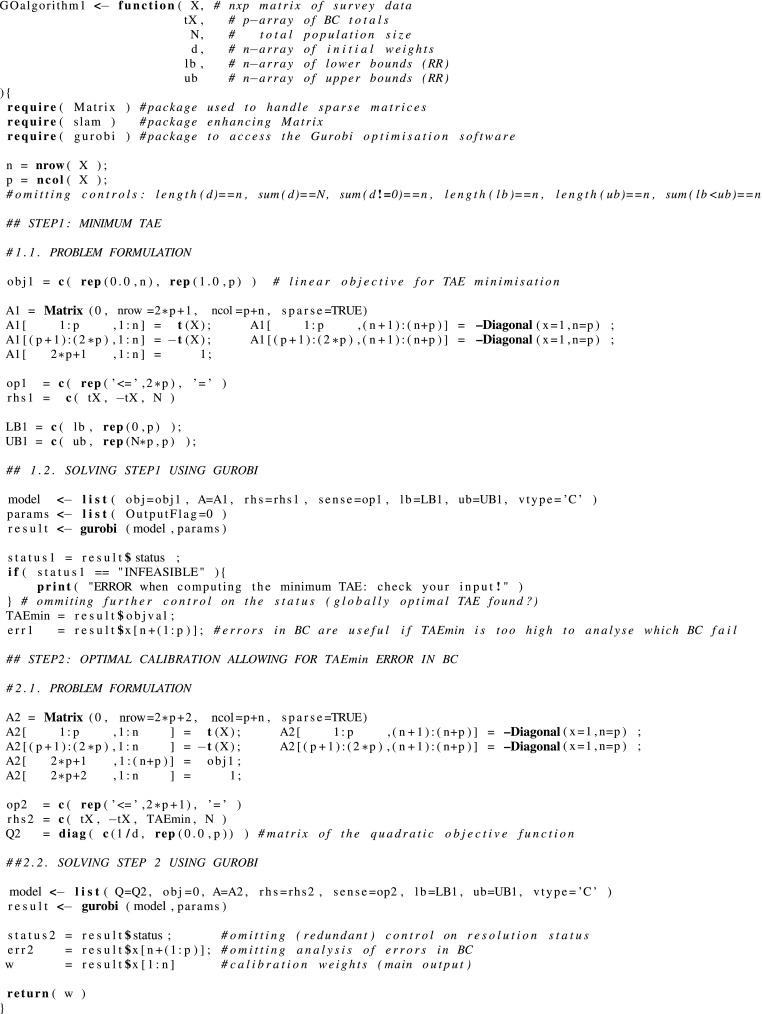
R Source code for Agorithm 1

## Appendix 1: Implementation details and R source code for Algorithm 1

We implemented our algorithms using the optimisation software Gurobi Optimization, Inc (2016). We called Gurobi functions from R Core Team (2016) using the R package “gurobi” provided by the software. The following additional R packages were used:

- Matrix: Sparse and Dense Matrix Classes and Methods^13^;
- slam: Sparse Lightweight Arrays and Matrices^14^.

R and the Matrix and slam packages were freely available under the terms of the GNU General Public Licence as published by the Free Software Foundation (version 2 or later), while access to Gurobi was granted under the terms of a free named-user academic licence. Figure 1 shows the R code for Algorithm 1 for demonstration purposes. Detail on the optimisation programs follows.

Assume given $\mathbf {X}$ survey data, $\mathbf {d}$ initial weights, $\mathbf {t}_\mathbf {x}$ array of BC, *N* the population size, and ${\mathbf {l}}, {\mathbf {u}}$ RR on weights.

*Minimum total absolute error in BC.* The minimum total absolute error achievable in BC is computed by solving (10) in the compact linear form (14). Note that the known population size is added to BC as additional linear constraint: ${\mathbf {1}}'\mathbf {w}= N$.

14 $$\begin{aligned} \begin{array}{rl} TAE^*= \min \nolimits _{\mathbf {w},\,\varvec{\tilde{\varepsilon }}} &{} \left( \varvec{0}', \varvec{1}' \right) \; \begin{pmatrix}\mathbf {w}\\ \varvec{\tilde{\varepsilon }}\end{pmatrix}\\ s.t.&{} \mathbf {A_1} \begin{pmatrix}\mathbf {w}\\ \varvec{\tilde{\varepsilon }}\end{pmatrix}\; \mathbf {op_1} \; \mathbf {rhs_1}\;,\\ &{} {{\mathbf {l}}}{{\mathbf {b}}} \le \begin{pmatrix}\mathbf {w}\\ \varvec{\tilde{\varepsilon }}\end{pmatrix} \le {{\mathbf {u}}}{{\mathbf {b}}}\;, \end{array} \end{aligned}$$

where $$\mathbf {A_1} = \begin{pmatrix} \mathbf {X}' &{} - {{\mathbf {I}}}{{\mathbf {d}}}\\ -\mathbf {X}' &{} - {{\mathbf {I}}}{{\mathbf {d}}}\\ {\mathbf {1}}' &{} {\mathbf {0}}' \end{pmatrix}$$, $\mathbf {op_1}$ is an array of operators with 2*p* times “$\le$” followed by a “$=$,” $$\mathbf {rhs_1} = \begin{pmatrix} \mathbf {t}_\mathbf {x}\\ -\mathbf {t}_\mathbf {x}\\ N \end{pmatrix}$$, $${{\mathbf {l}}}{{\mathbf {b}}}=\begin{pmatrix} {\mathbf {l}}\\ {\mathbf {0}} \end{pmatrix}$$, and $${{\mathbf {u}}}{{\mathbf {b}}}=\begin{pmatrix} {\mathbf {u}}\\ \infty \cdot {\mathbf {1}} \end{pmatrix}$$.

*Optimal calibration allowing for minimum error in BC.* The calibration problem is solved allowing for the minimum TAE error achievable in BC (determined in the previous step). The resulting quadratic optimisation problem, imposing the known population size as additional constraint, is summarised in (15).

15 $$\begin{aligned} \begin{array}{rl} \mathbf {w}^{Cal}= \arg \min _\mathbf {w}&{} \left( \mathbf {w}, \varvec{\tilde{\varepsilon }}\right) {\mathbf {Q}} \begin{pmatrix}\mathbf {w}\\ \varvec{\tilde{\varepsilon }}\end{pmatrix} + \left( \varvec{0}', \varvec{0}' \right) \begin{pmatrix}\mathbf {w}\\ \varvec{\tilde{\varepsilon }}\end{pmatrix}\\ s.t.&{} \mathbf {A_2} \begin{pmatrix}\mathbf {w}\\ \varvec{\tilde{\varepsilon }}\end{pmatrix}\; \mathbf {op_2} \; \mathbf {rhs_2}\;,\\ &{} {{\mathbf {l}}}{{\mathbf {b}}} \le \begin{pmatrix}\mathbf {w}\\ \varvec{\tilde{\varepsilon }}\end{pmatrix} \le {{\mathbf {u}}}{{\mathbf {b}}}\;, \end{array} \end{aligned}$$

where ${\mathbf {Q}}$ is a diagonal matrix with the inverse of $\mathbf {d}$ followed by ${\mathbf {0}}$ in the diagonal, $$\mathbf {A_2} = \begin{pmatrix} \mathbf {X}' &{} \quad - {\mathbf {I}}{\mathbf {d}}\\ -\mathbf {X}' &{} \quad - {\mathbf {I}}{\mathbf {d}}\\ \mathbf {0}' &{} \mathbf {1}'\\ \mathbf {1}' &{} \mathbf {0}' \end{pmatrix}$$, $\mathbf {op_2}$ is an array of operators with $2p+1$ times “$\le$” followed by a “$=$,” $$\mathbf {rhs_2} = \begin{pmatrix} \mathbf {t}_\mathbf {x}\\ -\mathbf {t}_\mathbf {x}\\ TAE^*\\ N \end{pmatrix}$$, and ${\mathbf {l}}{\mathbf {b}},{\mathbf {u}}{\mathbf {b}}$ are defined in (14).

## Appendix 2: Further validation

A further validation was performed using the 20 age by economic activity groups considered for validation in the main text, additionally cross-tabulated by region (a total of 180 counts to be estimated). Table 4 shows the validation results, which overall are consistent with Table 3. The gain in performance of our approach (Ex1–Ex3) with respect to HSE is more significant that in that table, possibly in part because age totals disaggregated by region are not used to build the HSE weights, and fundamentally because our approach allowed to optimally incorporate very detailed relevant auxiliary information to the calibration weights, which is exploited by the resulting estimates.

**Table 4:** Age by economic activity by region estimation (180 counts): average standard deviation (SD) over all estimates, and validation errors

	SD	TAE	TAE (%)	TRE (%)	RMSE
HSE	279,295.2	5,073,091.0	12.06	8936.7	44,071.9
Ex1	178,523.2	3,270,789.0	7.77	7883.2	26,536.4
Ex2	179,533.4	3,285,067.0	7.81	7884.3	26,876.9
Ex3	178,101.0	3,262,183.0	7.75	7892.7	26,481.1

Experiments Ex1–Ex3 only control for errors in BC, but do not use the validation table for calibrating the survey weights

## Footnotes

1 The case of a known group total $\mathbf {t}_\mathbf {G}$ is a particular case of BC in the form $\mathbf {1}_G' \mathbf {w}- \mathbf {t}_\mathbf {G}= {\mathbf {0}}$.

2 The case with no RR is a particular case in which ${\mathbf {l}}= {\infty } \cdot \mathbf {1}= - {\mathbf {u}}$ .

3 Alternatively used formulations of the calibration problem computing the relative change in weights are equivalent to (1), where in that case the unknown is ${\mathbf {g}}$ defined by ${g}_i = w_i/d_i, 1 \le i \le n$.

4 In the case of known totals and initial weights consistent with those totals ($\varvec{1}' \mathbf {w}= \varvec{1}' \mathbf {d}$ constant), the minimisation of the Chi-square distance simplifies to minimising $\mathbf {w}' \mathbf {D}^{-1} \mathbf {w}$.

5 If setting to $\pm \infty$ either of the RR vectors, we obtain the same questions for the other RR vector.

6 For any *x*, *y* real numbers, $|x|\le y$ is equivalent to $x\le y$ and $-x\le y$

7 Assuming independent identically distributed random errors in a linear system, the $\ell _1$-norm penalisation gives the MLE for a Laplacian distribution of errors, whereas the $\ell _2$- and $\ell _\infty$-norm penalisations give the MLE for Gaussian and uniform error distributions, respectively (Boyd and Vandenberghe 2004).

8 25 adults not having a valid economic activity were discarded.

9 Ages 0–4, 5–9, 10–15, 16–24, 25–34, 35–44, 45–54, 55–64, 65–74, 75+.

10 Ages 0–4, 5–7, 8–9, 10–14, 15, 16–17, 18–19, 20–24, 25–29, 30–34, 35–39, 40–44, 45–49, 50–54, 55–59, 60–64, 65–69, 70–74, 75–79, 80–84, 85+.

11 Ages 16–24, 25–34, 35–49, 50–64, 65+.

12 The HSE 2012 sample does not contain females in the North East aged 15.
