# Supplementary material for: A global optimisation approach to range-restricted survey calibration
Source: Stat Comput. 2017 Mar 21;28(2):427–39. doi: 10.1007/s11222-017-9739-5 (PMC6956878; doi:10.1007/s11222-017-9739-5)
Supplement: Supplementary file 1 — Supplementary material 1 (pdf 43 KB) [file 11222_2017_9739_MOESM1_ESM.pdf]

Noname manuscript No.
(will be inserted by the editor)

A global optimisation approach to range-restricted survey calibration
Supplementary material: benchmark and validation tables

Feran Espuny-Pujol · Karyn Morrissey · Paul Williamson

The following tables were used as benchmark constraints (BC) in experiments Ex1-Ex3:

- Tables 1 and 2 were imposed as exact BC;
- Table 3 was used as BC allowing for errors.

The following tables were used only for validating the estimation of non-BC counts with weights calibrated using BC:

- Table 4 was used for validation;
- Table 5 was used for additional validation.

Table 1 Age-gender totals for England (20 counts).

Age	M	F
0-4	1,696,798	1,619,067
5-9	1,519,838	1,450,348
10-15	1,891,048	1,805,476
16-24	2,976,361	2,925,446
25-34	3,512,442	3,561,610
35-44	3,653,241	3,733,671
45-54	3,578,713	3,658,655
55-64	3,017,466	3,116,743
65-74	2,166,363	2,347,583
75+	1,591,512	2,237,550

Ferran Espuny-Pujol (E-mail: F.Espuny-Pujol@uea.ac.uk)
Health Economics Group, Norwich Medical School, University of East Anglia, Norwich, UK

Karyn Morrissey (E-mail: K.Morrissey@exeter.ac.uk)
European Centre for Environment and Human Health, University of Exeter Medical School, Truro, UK

Paul Williamson (E-mail: P.Williamson@liverpool.ac.uk)
Department of Geography and Planning, University of Liverpool, Liverpool, UK

Table 2 Region totals for England (9 counts).

Region	Total
North East	2,550,818
North West	6,927,820
Yorkshire and The Humber	5,185,677
East Midlands	4,442,192
West Midlands	5,509,535
East of England	5,748,605
London	8,073,700
South East	8,446,500
South West	5,175,084

Table 3 Age-gender-region totals for England (378 counts).

Age	Gender	NE	NW	YH	EM	WM	EE	L	SE	SW
0-4	M	76,766	221,109	167,756	137,688	181,661	184,795	302,343	273,530	151,150
0-4	F	72,944	210,650	160,483	132,303	172,911	176,161	288,816	260,176	144,623
5-7	M	42,703	123,516	93,165	77,531	102,619	103,026	153,289	153,164	85,033
5-7	F	40,952	117,510	89,594	73,939	98,105	97,943	147,724	145,979	80,930
8-9	M	27,232	77,613	58,401	48,855	64,590	65,553	92,476	96,501	54,571
8-9	F	25,322	73,371	56,130	47,079	61,562	61,745	89,187	91,623	51,653
10-14	M	73,844	210,050	154,816	134,395	173,525	175,927	232,728	257,312	149,257
10-14	F	70,836	200,764	149,621	128,358	165,290	167,233	223,437	245,531	142,847
15	M	16,297	44,563	33,357	28,799	36,214	36,667	48,364	53,008	31,925
15	F	14,839	42,586	31,805	26,611	34,749	35,827	44,906	50,165	30,071
16-17	M	32,223	90,415	66,031	57,438	72,819	74,568	94,607	106,122	62,748
16-17	F	30,701	84,640	62,838	54,711	70,264	70,366	90,408	101,349	60,195
18-19	M	33,292	86,020	65,864	53,695	69,036	64,186	87,826	96,073	59,742
18-19	F	32,558	82,816	64,168	51,534	65,499	60,821	86,335	91,252	57,364
20-24	M	88,680	231,128	180,085	144,416	182,085	167,638	298,850	251,510	159,264
20-24	F	87,281	232,574	182,277	144,431	179,873	165,326	308,171	250,479	157,215
25-29	M	79,873	229,173	168,884	135,519	178,895	175,714	407,688	255,160	149,893
25-29	F	82,300	231,991	172,908	138,656	180,049	179,761	417,793	261,046	150,427
30-34	M	73,827	211,964	159,338	131,750	169,012	177,606	400,447	261,504	146,195
30-34	F	75,967	214,577	159,245	133,390	171,751	182,132	392,219	270,617	146,781
35-39	M	76,224	220,020	167,249	142,586	178,538	189,888	334,129	281,137	157,349
35-39	F	79,807	227,042	168,557	146,987	179,365	194,927	326,733	292,002	162,059
40-44	M	88,693	252,089	188,771	164,216	199,987	211,892	302,361	315,009	183,103
40-44	F	93,366	259,745	192,042	169,388	204,118	218,147	304,373	325,332	189,681
45-49	M	95,364	255,509	189,002	167,249	199,246	214,047	271,414	321,604	190,260
45-49	F	99,534	263,935	191,016	170,854	202,604	219,257	281,819	327,534	196,521
50-54	M	88,588	229,157	169,817	147,740	173,659	188,016	226,278	280,636	171,127
50-54	F	91,759	233,412	172,211	149,307	175,637	190,385	232,262	283,754	176,854
55-59	M	78,800	202,962	151,354	133,442	158,214	166,405	180,085	243,221	156,613
55-59	F	81,271	204,365	152,301	134,955	159,765	172,565	189,525	249,464	164,353
60-64	M	82,006	217,010	159,501	143,690	164,526	180,440	164,332	259,936	174,929
60-64	F	85,567	220,144	164,127	146,184	169,096	190,322	176,139	272,201	184,399
65-69	M	62,102	165,559	121,602	113,095	135,951	142,797	120,018	203,673	143,801
65-69	F	65,484	174,238	129,300	117,579	142,175	149,929	134,735	217,567	151,653
70-74	M	50,911	133,114	97,887	86,869	106,656	112,336	99,469	158,979	111,544
70-74	F	58,424	148,859	111,190	93,805	117,072	122,627	114,315	176,965	121,666
75-79	M	39,194	99,270	74,685	66,087	80,928	89,931	78,053	126,246	87,923
75-79	F	48,316	122,560	92,613	77,231	96,612	105,198	95,237	150,221	103,970
80-84	M	25,171	64,747	49,008	44,036	54,391	61,825	52,507	87,824	62,084
80-84	F	35,988	93,193	70,154	60,548	75,212	82,921	73,995	119,970	83,966
85+	M	15,706	42,386	33,294	30,486	35,938	43,389	37,297	63,482	45,624
85+	F	30,106	81,474	63,230	54,760	69,336	78,366	71,010	117,642	83,721

Table 4 Age by economic activity totals for England (20 counts).

	Age 16-24	Age 25-34	Age 35-49	Age 50-64	Age 65+
in-employment	3,109,928	5,616,663	9,179,826	6,364,788	871,509
unemployed	711,225	428,631	514,897	302,440	17,233
retired	2,936	3,649	24,596	1,622,139	7,194,505
other inactive	2,077,718	1,025,109	1,524,362	1,225,441	259,761

Table 5 Age by economic activity by region totals for England (180 counts).

Age	EconAct	NE	NW	YH	EM	WM	EE	L	SE	SW
16-24	in-employment	150,984	423,064	320,578	270,772	320,015	352,344	434,924	513,289	323,958
16-24	unemployed	43,744	104,991	81,600	61,057	86,599	66,900	117,843	93,454	55,037
16-24	retired	136	414	276	194	320	331	521	463	281
16-24	other inactive	109,871	279,124	218,809	174,202	232,642	183,330	412,909	289,579	177,252
25-34	in-employment	238,594	690,162	509,411	429,921	531,750	581,694	1,279,820	864,949	490,362
25-34	unemployed	25,361	61,670	47,001	32,621	52,504	35,995	98,575	47,732	27,172
25-34	retired	181	478	322	253	384	369	764	555	343
25-34	other inactive	47,831	135,395	103,641	76,520	115,069	97,155	238,988	135,091	75,419
35-49	in-employment	425,824	1,188,208	892,917	799,833	937,688	1,045,254	1,402,550	1,574,560	912,992
35-49	unemployed	29,300	70,452	54,669	40,771	61,461	48,733	107,751	65,105	36,655
35-49	retired	1,317	3,726	2,543	2,167	2,597	2,564	3,398	3,690	2,594
35-49	other inactive	76,547	215,954	146,508	118,509	162,112	151,607	307,130	219,263	126,732
50-64	in-employment	309,071	825,600	627,087	572,625	657,769	758,594	772,326	1,129,026	712,690
50-64	unemployed	17,959	40,709	31,238	25,091	35,203	31,650	50,834	43,962	25,794
50-64	retired	98,072	239,571	178,980	153,220	170,989	183,891	155,637	258,174	183,605
50-64	other inactive	82,889	201,170	132,006	104,382	136,936	113,998	189,824	158,050	106,186
65+	in-employment	28,025	98,387	71,986	70,573	90,596	112,209	114,924	172,442	112,367
65+	unemployed	622	1,872	1,308	1,297	1,713	1,947	3,367	3,200	1,907
65+	retired	388,471	986,731	743,268	652,230	792,127	850,121	713,714	1,210,978	856,865
65+	other inactive	14,284	38,410	26,401	20,396	29,835	25,042	44,631	35,949	24,813
